# Replication stress inhibits synthesis of histone mRNAs in yeast by removing Spt10p and Spt21p from the histone promoters

**DOI:** 10.1016/j.jbc.2021.101246

**Published:** 2021-09-25

**Authors:** Madhura Bhagwat, Shreya Nagar, Pritpal Kaur, Riddhi Mehta, Ivana Vancurova, Ales Vancura

**Affiliations:** Department of Biological Sciences, St John's University, Queens, New York, USA

**Keywords:** replication stress, histone mRNA, chromatin, transcription, mRNA decay, promoter, ChIP, chromatin immunoprecipitation, DDC, DNA damage checkpoint, DRC, DNA replication checkpoint, HU, hydroxyurea, MBF, MCB-binding factor, NEG, negative regulatory, PIC, preinitiation complex, RT-qPCR, reverse transcription-quantitative PCR, SBF, SCB-binding factor

## Abstract

Proliferating cells coordinate histone and DNA synthesis to maintain correct stoichiometry for chromatin assembly. Histone mRNA levels must be repressed when DNA replication is inhibited to prevent toxicity and genome instability due to free non-chromatinized histone proteins. In mammalian cells, replication stress triggers degradation of histone mRNAs, but it is unclear if this mechanism is conserved from other species. The aim of this study was to identify the histone mRNA decay pathway in the yeast *Saccharomyces cerevisiae* and determine the mechanism by which DNA replication stress represses histone mRNAs. Using reverse transcription-quantitative PCR and chromatin immunoprecipitation–quantitative PCR, we show here that histone mRNAs can be degraded by both 5′ → 3′ and 3′ → 5′ pathways; however, replication stress does not trigger decay of histone mRNA in yeast. Rather, replication stress inhibits transcription of histone genes by removing the histone gene–specific transcription factors Spt10p and Spt21p from histone promoters, leading to disassembly of the preinitiation complexes and eviction of RNA Pol II from histone genes by a mechanism facilitated by checkpoint kinase Rad53p and histone chaperone Asf1p. In contrast, replication stress does not remove SCB-binding factor transcription complex, another activator of histone genes, from the histone promoters, suggesting that Spt10p and Spt21p have unique roles in the transcriptional downregulation of histone genes during replication stress. Together, our data show that, unlike in mammalian cells, replication stress in yeast does not trigger decay of histone mRNAs but inhibits histone transcription.

Proliferating cells need to maintain a delicate balance between histone and DNA synthesis to ensure correct stoichiometric amounts for chromatin assembly and avoid genome instability (1, 2). Treatment of cells with genotoxic agents that damage DNA and inhibit DNA replication decreases histone mRNAs to avoid accumulation of free nonchromatinized histone proteins, which are toxic to the cell (3, 4, 5). While it is well established that DNA replication correlates with histone mRNA levels, and that activation of DNA damage checkpoint (DDC) or DNA replication checkpoint (DRC) represses histone mRNA levels (3, 4, 5), the corresponding mechanisms are not fully understood.

Budding yeast possesses two genes encoding each of the four major core histones. The genes are organized into four loci, each containing two histone genes divergently transcribed from a central promoter. Two loci, *HHT1-HHF1* and *HHT2-HHF2*, encode identical H3 and H4 proteins. The other two loci, *HTA1-HTB1* and *HTA2-HTB2*, encode almost-identical H2A and H2B proteins. The expression of histone genes is activated by Spt10p, Spt21p, SCB-binding factor (SBF), and MCB-binding factor (MBF) and repressed by the HIR complex (1, 2, 6, 7, 8). Spt10p is a histone gene-specific transcription factor that binds to the upstream-activation sequences of all histones (7, 9, 10). During the S phase, Spt10p associates with Spt21p (11, 12). The protein level of Spt21p is cell cycle regulated; Spt21p is degraded during the G1 and G2/M phases and accumulates only during the S phase, when it binds to histone gene promoters and recruits Gcn5p histone acetyltransferase (8). Activation of histone gene promoters during the S phase also requires the switch/sucrose nonfermentable chromatin remodeling complex (13). In addition, the activation of histone genes early in the S phase is mediated by the SBF and MBF complexes (14). SBF and MBF recognize sites in G1/S promoter regions, called SCB (Swi4/6 cell cycle box) and MCB (*MluI* cell cycle box), respectively. The SBF complex consists of Swi4p and Swi6p. The MBF complex consists of Mbp1p and Swi6p. Mbp1p and Swi4p proteins are the DNA-binding components of MBF and SBF, respectively, while Swi6p plays a regulatory role in both complexes. Outside of the S phase, Spt10p facilitates recruitment of the histone regulatory (HIR) complex to the negative regulatory (NEG) elements in the histone promoters to establish their repression (1, 2, 8). The HIR complex and Spt10p recruit a myriad of additional factors, including histone chaperones Asf1p and Rtt106, chromatin boundary protein Yta7p, and the remodel structure of chromatin complex, which together repress histone genes transcription. (1, 2). The transcriptional repression of histone genes at the end of the S phase or during replication stress induced by hydroxyurea (HU) treatment operates at all 4 histone loci and, except for *HTA2–HTB2*, requires the HIR complex, Rtt106p, and Asf1p (15, 16, 17). Rtt106p is recruited to histone promoters in a histone-dependent manner, indicating that the repressive chromatin structure at the histone promoters requires free histones, which accumulate when DNA replication stops (17, 18).

The level of mRNA is determined by its synthesis and degradation rates. mRNA degradation is an important factor regulating gene expression and complements transcriptional regulation by enabling cells to rapidly vary the levels of existing transcripts. Degradation of mRNA can occur by two general pathways, in 3′ to 5′ direction and in 5′ to 3′ direction (19). Previous studies have suggested that DDC/DRC activation affects both histone transcription and histone mRNA stability in yeast (20, 21, 22, 23, 24, 25, 26). The aim of this study was to identify the histone mRNA decay pathway and determine the mechanism by which DDC/DRC activation represses histone mRNAs. We show here that histone mRNAs are degraded by both 5′ to 3′ and 3′ to 5′ pathways; however, DDC/DRC activation does not trigger decay of histone mRNA. Rather, replication stress inhibits transcription of histone genes by removing Spt10p and Spt21p from histone promoters. The replication stress–induced disassembly of the preinitiation complex (PIC) at the histone promoters is facilitated by Rad53p and Asf1p.

## Results

### Genotoxic stress represses histone mRNA levels

**Figure 1:**
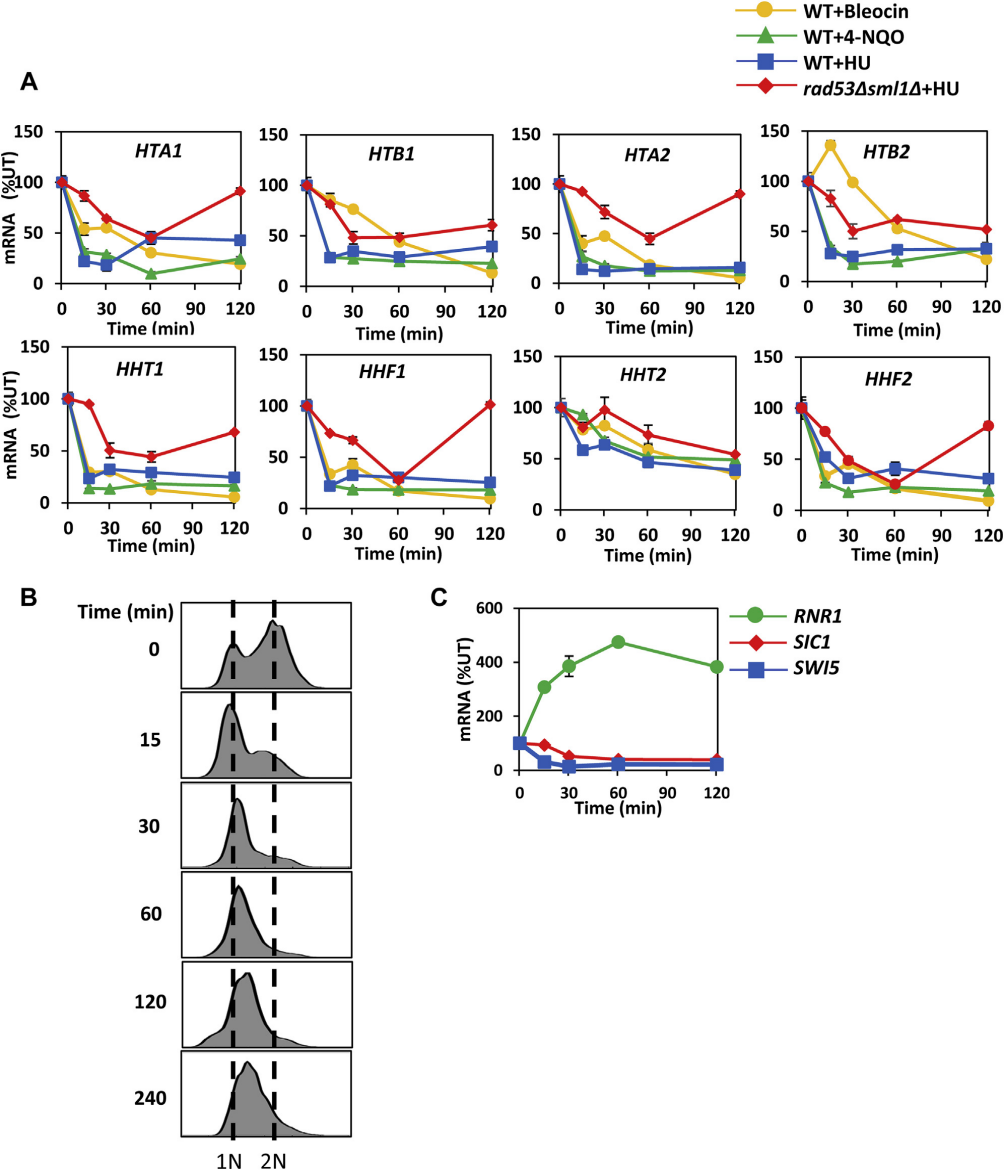
**Genotoxic stress represses histone mRNA levels.***A*, time course of histone mRNA levels in WT cells (W303-1a) treated with bleocin at 1.0 μg/ml, 4-nitroquinoline 1-oxide (4-NQO) at 1.0 μg/ml, and hydroxyurea (HU) at 200 mM. Histone mRNA levels in *rad53*Δ*sml1*Δ (LG606) cells treated with 200 mM HU are also shown. The results are expressed relative to the value for the WT or *rad53*Δ*sml1*Δ strain grown in the absence of genotoxic chemicals. *B* and *C*, asynchronously growing WT cells (W303-1a) treated with 200 mM HU arrest in the S phase. *B*, cells were fixed, and the DNA content was measured by flow cytometry. *C*, mRNA levels of *RNR1*, *SIC1*, and *SWI5*.

To determine whether DDC/DRC activation represses mRNA levels of all histones, we utilized bleocin, 4-nitroquinoline 1-oxide, and HU. Bleocin belongs to the antibiotic bleomycin family and causes DNA double-strand breaks (27). 4-Nitroquinoline 1-oxide mimics the effect of UV light and forms DNA adducts (28). HU is an inhibitor of ribonucleotide reductase, decreases dNTP levels, and slows down progression of the replication fork. Treatment of WT cells with either chemical rapidly decreased mRNA levels of all histone genes (Fig. 1*A*). This effect depended on Rad53p because the repression of histone mRNA levels was significantly attenuated in *rad53*Δ*sml1*Δ cells (Fig. 1*A*). This is in agreement with our previous results that showed histone mRNA levels reduced in an Rad53p-dependent manner in *rad52*Δ cells (29). *RAD52* is required for DNA double-strand break repair and homologous recombination. Inactivation of *RAD52* makes cells unable to repair DNA strand breaks and activates DDC (30).

To eliminate the possibility that the HU-mediated repression of histone mRNAs is not due to the cell cycle arrest outside of the S phase when histones are not transcribed, we determined distribution of cells throughout the cell cycle during HU treatment of asynchronous cells (Fig. 1*B*). The results show that HU arrests cells early in the S phase, in agreement with other studies (31, 32). In addition, mRNA levels of *SIC1*, *RNR1*, and *SWI5* during the HU treatment of asynchronous cells also indicate that cells accumulate in the S phase (Fig. 1*C*). mRNA levels of *SIC1*, *RNR1*, and *SWI5* peak in early G1, G_1_/S transition, and M phases of the cell cycle, respectively (33, 34, 35).

**Figure 2:**
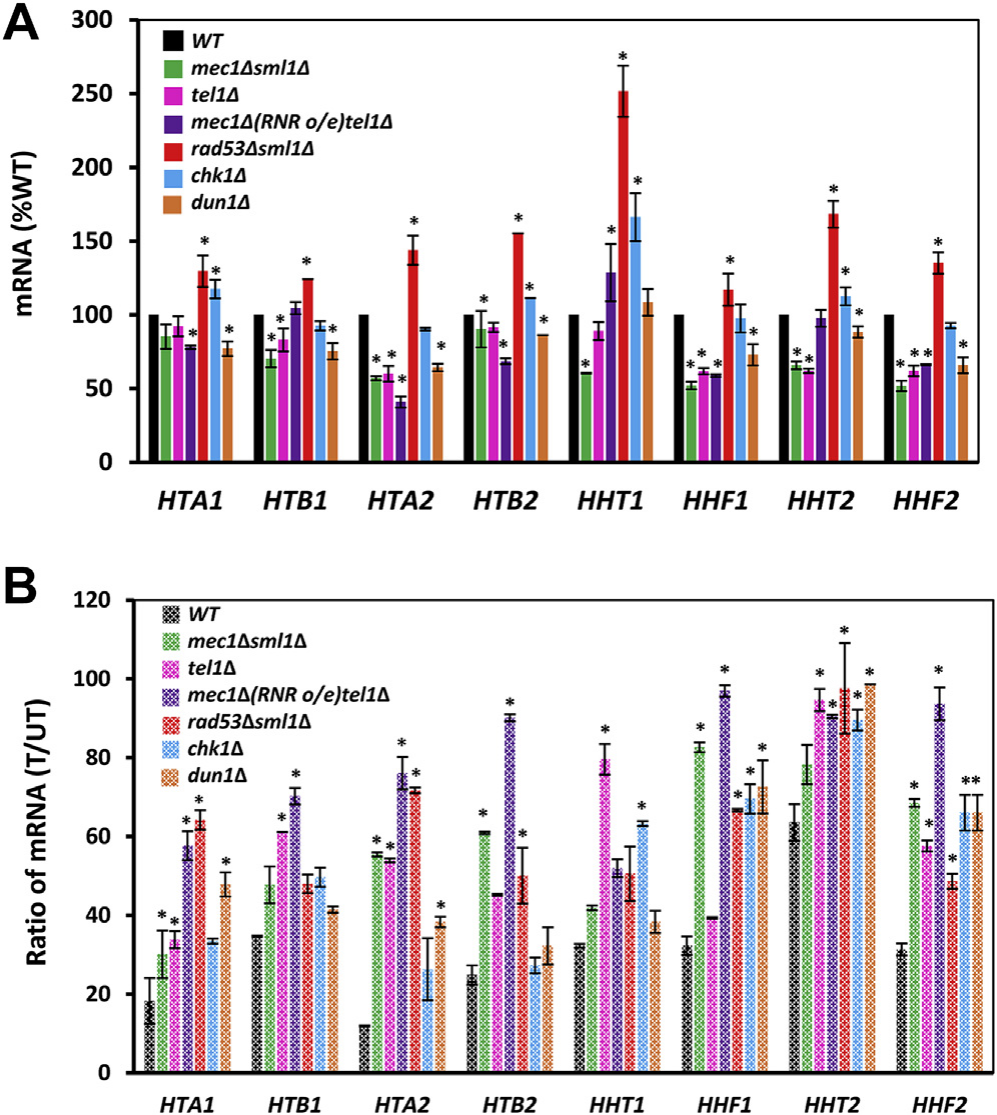
**Genotoxic stress represses histone mRNA levels in checkpoint kinase–dependent manner.***A*, histone mRNA levels in WT (W303-1a), *mec1*Δ*sml1*Δ (SN117), *tel1*Δ (SN159), *mec1*Δ*tel1*Δ [*RNR1* o/e] (MB181), *rad53*Δ*sml1*Δ (LG606), *chk1*Δ (SN136), and *dun1*Δ (PB119) cells. The results are expressed relative to the value for the WT strain. *B*, histone mRNA levels remaining after 30-min HU treatment, calculated as a ratio of mRNA levels in treated (T)/untreated (UT) samples for each individual strain. *A* and *B*, the experiments were repeated three times, and the results are shown as the means ± SD. Values that are statistically different (*p* < 0.05) from the WT cells are indicated by an *asterisk*. HU, hydroxyurea.

To systematically determine whether other checkpoint kinases are required for replication stress–induced repression of histone mRNAs, we first determined histone mRNA levels in WT, *mec1*Δ*sml1*Δ, *tel1*Δ, *mec1*Δ*tel1*Δ [*RNR1*], *rad53*Δ*sml1*Δ, *chk1*Δ, and *dun1*Δ cells; *mec1*Δ and *rad53*Δ cells are viable only if harboring the *sml1*Δ mutation or overexpress *RNR1* gene (36). *rad53*Δ*sml1*Δ cells displayed the highest mRNA levels for all histones (Fig. 2*A*). This is in agreement with a recent study demonstrating that under physiological conditions, Rad53p regulates histone mRNA levels by phosphorylating Spt21p (37). To assess the involvement of individual checkpoint kinases in HU-mediated repression of histone mRNAs, we calculated fractions of histone mRNAs remaining after 30 min of HU exposure (Fig. 2*B*). In WT cells, the level of repression varied for individual histone genes, ranging from about 10% for *HTA2*, to about 60% for *HHT2*, perhaps suggesting that individual histone genes have partly unique modes of regulation. Cumulatively, the results show that all checkpoint kinases play a role in repressing histone mRNA levels in response to HU treatment, working either redundantly through the same mechanism or individually through different mechanisms. Because *rad53*Δ*sml1*Δ cells displayed the highest mRNA levels for all histones (Fig. 2*A*) and a significant defect in HU-mediated repression of histone mRNAs (Fig. 2*B*), we conclude that Rad53p plays an important role in DDC/DRC-mediated regulation of histone mRNAs.

### Histone mRNAs are degraded by canonical 5′ to 3′ and 3′ to 5′ pathways

**Figure 3:**
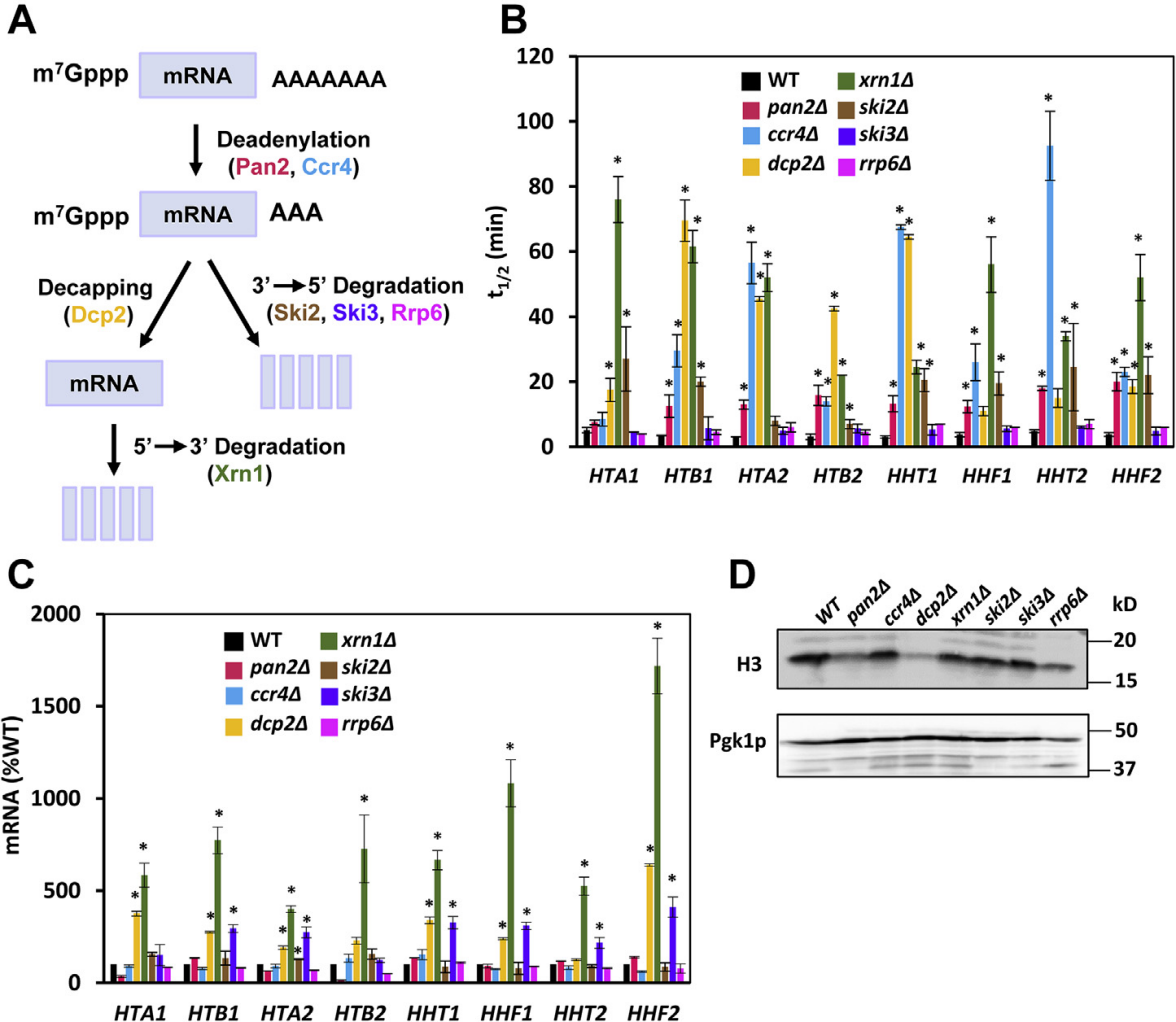
**Histone mRNAs are degraded by both 5′**→**3′ and 3′**→**5′ pathways.***A*, a model depicting the 5′→3′ and 3′→5′ pathways of mRNA decay and the corresponding enzymes. *B*, half-lives of histone mRNAs (t_1/2_), (*C*) steady-state histone mRNA levels, and (*D*) histone H3 protein levels in WT (W303-1a), *pan2*Δ (MB123), *ccr4*Δ (SM096), *dcp2*Δ (yRP2859), *xrn1*Δ (MB115), *ski2*Δ (MB133), *ski3*Δ (MB109), and *rrp6*Δ (MB120) cells. *B* and *C*, the experiments were repeated three times, and the results are shown as the means ± SD. Values that are statistically different (*p* < 0.05) from the WT cells are indicated by an *asterisk*. *C*, the results are expressed relative to the value for the WT strain. *D*, Western blot was performed three times, and representative results are shown. Pgk1p served as a loading control.

Degradation of mRNA occurs by two general pathways, in 3′ to 5′ direction or in 5′ to 3′ direction (Fig. 3*A*) (19). Both pathways are initiated by deadenylation, which results in shortening of the 3′ poly(A) tail. Deadenylation is carried out by the Pan2p–Pan3p complex as well as by the Ccr4p–Pop2p–Not complex. Subsequently, the cap structure (5′-m^7^GpppN) is removed by the decapping complex Dcp1p/Dcp2p, and mRNA is degraded in 5′ to 3′ direction by the major cytoplasmic enzyme Xrn1p. Interestingly, Xrn1p is a substrate of Rad53p (38). Alternatively, deadenylated mRNAs can be degraded from their 3′ ends by the exosome, a multimeric complex with 3′ to 5′ exoribonuclease activity; Ski2p, Ski3p, and Rrp6p are subunits of the exosome (19).

Yeast histone mRNAs are quite unstable and have short half-lives under normal conditions (39, 40, 41); however, it is not known whether they are degraded in 3′ to 5′ direction, or in 5′ to 3′ direction. To investigate the pathway responsible for histone mRNA decay, we inhibited transcription with thiolutin and determined half-lives of histone mRNAs by following mRNA levels of individual histones by reverse transcription-quantitative PCR (RT-qPCR). We used primers that amplify the 3′ end of the translated sequence and adjacent not-translated portion and can differentiate between the two genes encoding each histone. Our results showed that deadenylation carried out by both Pan2p–Pan3p and Ccr4p–Pop2p–Not complexes play a role in regulating histone mRNA decay, with the Ccr4p–Pop2p–Not complex playing the more dominant role (Fig. 3*B*). Inactivation of the decapping complex Dcp1p–Dcp2p by *dcp2*Δ mutation also significantly stabilized histone mRNAs. After decapping, majority of histone mRNAs were degraded by the 5′ to 3′ exonuclease Xrn1p, as indicated by greatly stabilized histone mRNAs in *xrn1*Δ cells. In comparison, the cytosolic exosome played a lesser role in degradation of histone mRNAs than Xrn1p, as suggested by histone mRNA half-lives in *ski2*Δ cells. Ski2p is required for cytosolic function of the exosome (19). Conversely, inactivation of *RRP6*, required for the function of the exosome in the nucleus, affected stability of the histone mRNAs only slightly (Fig. 3*B*). The half-lives of individual histone mRNAs were between 3 and 5 min (Fig. 3*B*). Short half-lives are typical for cell cycle–regulated mRNAs in organisms with short generation time such as *Saccharomyces cerevisiae* (39, 40, 41) because the corresponding mRNAs must accumulate and be degraded within a brief time window. We conclude that histone mRNAs are constitutively degraded by both 5′ to 3′ and 3′ to 5′ pathways, with Xrn1p making the major contribution in the 5′ to 3′ pathway.

Because inactivation of individual components of 5′ to 3′ and 3′ to 5′ decay pathways resulted in significantly elevated half-lives of histone mRNAs, we anticipated that the steady-state levels of histone mRNAs would be correspondingly increased. However, this prediction turned out to be correct only for *xrn1*Δ, *dcp2*Δ, and, to a lesser extent, *ski3*Δ mutants. The steady-state histone mRNA levels were almost unchanged in *ccr4*Δ and *pan2*Δ cells despite their significantly increased stability (Fig. 3*C*). We believe that this result can be explained by transcriptional buffering, where a defect in the mRNA decay pathway is compensated for by a decrease in transcription (42, 43, 44, 45). This possibility is in agreement with the notion that Xrn1p is required for transcriptional buffering and *xrn1*Δ cells accumulate mRNAs (43). Whether Dcp1p is also required for transcriptional buffering has not been determined yet. Despite the significantly elevated histone mRNA levels in *xrn1*Δ and *dcp2*Δ cells, the level of histone H3 protein in these mutants was not correspondingly increased (Fig. 3*D*). These results suggest that the histone mRNAs in *xrn1*Δ and *dcp2*Δ cells are not translating and are probably stored in P-bodies. This interpretation is in agreement with the observation that the size and number of P-bodies are elevated in *xrn1*Δ and *dcp2*Δ cells (46).

### DRC activation does not destabilize histone mRNAs

**Figure 4:**
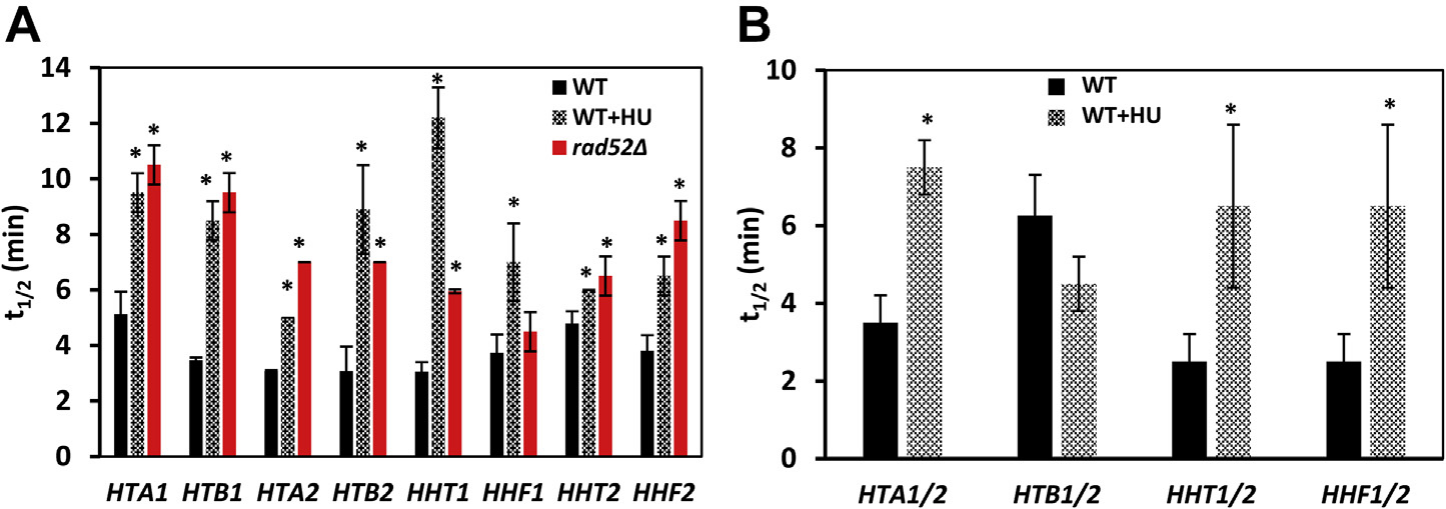
**Histone mRNAs are not destabilized by DRC activation.***A*, half-lives of individual histone mRNAs in WT (W303-1a) cells before and after hydroxyurea (HU) treatment (1 h) and in *rad52*Δ (LG731) cells were determined with primers that recognize individual histone mRNAs and amplify the 3′ ends of the translated sequences and adjacent not-translated sequences. *B*, half-lives of histone mRNAs in WT (W303-1a) cells before and after HU treatment (1 h) were determined with primers that amplify 5′ ends of transcripts of both genes for particular histone (*HTA1* and *HTA2*, *HTB1* and *HTB2*, *HHT1* and *HHT2*, and *HHF1* and *HHF2*). The experiments were repeated three times, and the results are shown as the means ± SD. Values that are statistically different (*p* < 0.05) from untreated WT cells are indicated by an *asterisk*. DRC, DNA replication checkpoint.

To determine whether activation of the DDC/DRC affects the stability of histone mRNAs, we incubated the cells for 60 min in the presence of 200 mM HU and determined histone mRNA half-lives. In an alternative approach, we determined histone mRNA half-lives in *rad52*Δ cells (Fig. 4*A*). Regardless of whether DDC/DRC was activated by treating cells with HU or by using the *rad52*Δ mutation, DDC/DRC activation did not destabilize histone mRNAs and thus cannot account for the decreased histone mRNA levels in cells treated with genotoxic chemicals (Fig. 1*A*). On the contrary, DDC/DRC activation resulted in somewhat increased half-lives of histone mRNAs (Fig. 4*A*). To confirm this result, in addition to primers that amplify 3′ ends of histone mRNAs, we also used primers that amplify 5′ ends of the translated sequences. However, these primers do not differentiate between the two transcripts of each histone and the reported half-lives (t_1/2_) represent cumulative stabilities of the two mRNAs (*HTA1* and *HTA2*, *HTB1* and *HTB2*, *HHT1* and *HHT2*, and *HHF1* and *HHF2*). With the exception of *HTB1/2*, the mRNA stabilities measured with these primers confirm that DDC/DRC activation does not destabilize histone mRNAs (Fig. 4*B*). DDC/DRC activation appears to slightly destabilize *HTB1/2*, in contrast to the results obtained with primers that amplify 3′ ends of histone mRNAs. However, the HU-triggered destabilization of *HTB1/2* mRNAs measured with primers that amplify 5′ ends of the translated sequences is relatively small and statistically not significant.

### DDC/DRC activation inhibits transcription of histone genes independently of SBF and MBF

Because DDC/DRC activation did not destabilize histone mRNAs, we next addressed whether DDC/DRC affects transcription of histone genes. The obvious candidates that link DDC/DRC and histone gene transcription are G_1_/S-specific transcription complexes SBF and MBF (1, 2). DDC/DRC activation induces Rad53p-dependent phosphorylation of Swi6p, resulting in downregulation of *CLN1* and *CLN2* transcription, and delayed G_1_ to S progression (47, 48). On the other hand, Rad53p phosphorylates and inactivates Nrm1p, a corepressor of MBF, resulting in the activation of MBF targets (49, 50). In addition, a systematic phosphoproteomics screen identified Swi6p, Swi4p, and Mbp1p as direct targets of Rad53p (51).

**Figure 5:**
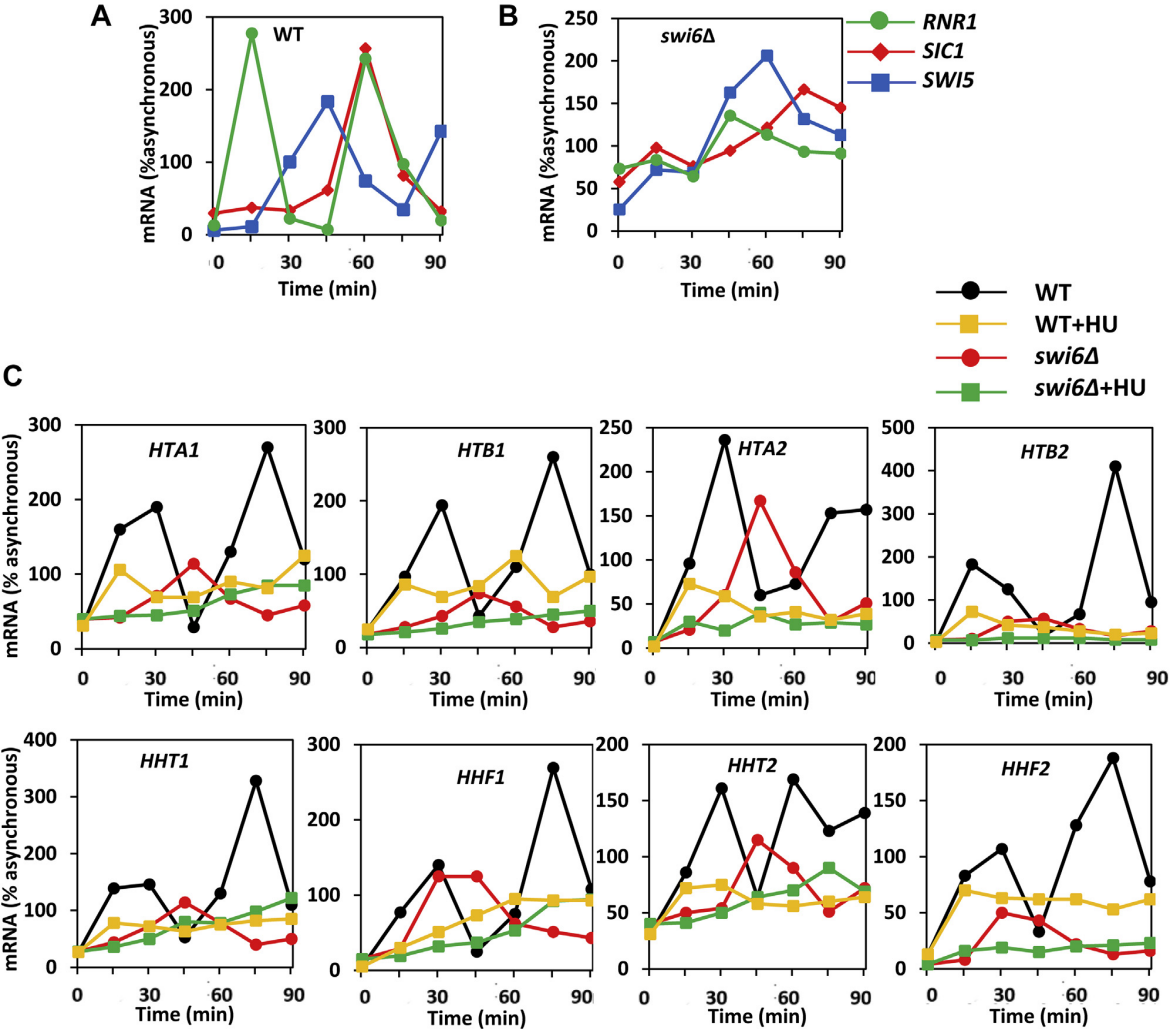
**DRC activation inhibits transcription of histone genes independently of SBF and MBF.***A*, WT (W303-1a) and (*B*) *swi6*Δ (DY5780) cells were grown in the YPD medium and synchronized with α-factor arrest and release. mRNA levels of *RNR1*, *SIC1*, and *SWI5* are expressed relative to the values for the corresponding asynchronous cells. *C*, WT (W303-1a) and *swi6*Δ (DY5780) cells were grown in the YPD medium and synchronized with α-factor arrest and release in the absence and presence of hydroxyurea (HU). mRNA levels are expressed relative to the values for the asynchronous WT cells. MBF, MCB-binding factor; SBF, SCB-binding factor.

Deletion of *SWI6* inactivates both MBF and SBF complexes and affects cell cycle progression by delaying the onset of the S phase (52). To determine the roles of MBF and SBF in DDC/DRC-mediated repression of histone genes, we measured histone mRNA levels during the cell cycle in WT and *swi6*Δ cells in the absence or presence of HU (Fig. 5). We reasoned that DDC/DRC activation would affect histone transcription in *swi6*Δ cells only if SBF or MBF complexes are not the only DDC/DRC targets involved in histone transcription. To determine whether in *swi6*Δ cells histones are transcribed during the S phase and whether the HU-triggered reduction in histone transcription occurs during the S phase, we also measured mRNA levels of *SIC1*, *RNR1*, and *SWI5*, markers for early G_1_, G_1_/S transition, and M phases of the cell cycle, respectively (33, 34, 35). After release from α-factor-mediated G_1_ arrest of the WT cells, the mRNA level of *RNR1* peaked first at 15 min, followed by *SWI5* at 45 min and *SIC1* at 60 min, indicating normal progression through S, G_2_/M, and G_1_ stages of the cell cycle (Fig. 5*A*). In *swi6*Δ cells, *RNR1*, *SWI5*, and *SIC1* peaked at 45 min, 60 min, and 75 min, respectively, indicating a delayed onset of the S phase (Fig. 5*B*). Expression of all histone genes in WT cells peaked at 15 to 30 min, corresponding to the S phase (Fig. 5*C*). The expression of histone genes in *swi6*Δ cells was delayed and attenuated in comparison with WT cells (Fig. 5*C*). However, histone mRNAs in *swi6*Δ cells still displayed a cell cycle–dependent profile, peaking during the S phase at 45 min, at the same time as *RNR1* (Fig. 5*B*). Importantly, DRC activation by HU in *swi6*Δ cells almost completely eliminated the accumulation of histone mRNAs during the S phase. Because both SBF and MBF complexes are inactivated in *swi6*Δ cells, we interpret these results to mean that DRC represses transcription of histone genes at least partly independently of SBF and MBF. The DRC-mediated repression of histone genes in the *swi6*Δ cells during the S phase is also clear for *HTA2* and *HTB2* genes. Unlike the other three histone gene pairs, the *HTA2–HTB2* promoter does not contain the NEG sequence, required for the assembly of the Hir complex and repression of histone genes outside of the S phase (1, 2). This observation suggests that DRC represses histone genes independently of the Hir complex assembled at the NEG sequence.

### DRC-triggered repression of histone mRNAs is attenuated in asf1Δ cells

**Figure 6:**
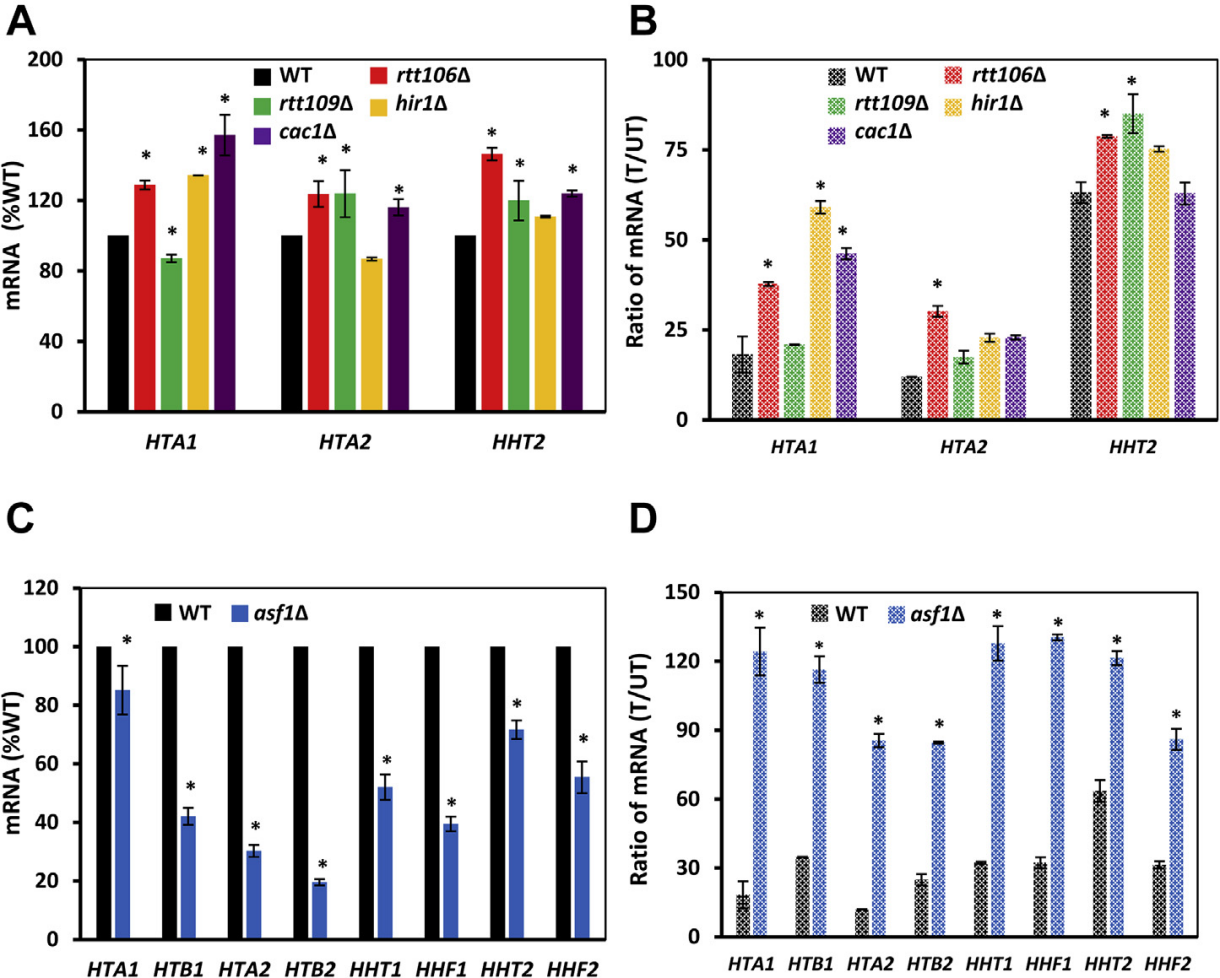
**The DRC-triggered repression of histone mRNAs is attenuated in *asf1*Δ cells.***A* and *C*, histone mRNA levels in WT (W303-1a), *rtt106*Δ (MZ642), *rtt109*Δ (MZ655), *hir1*Δ (MZ700), *cac1*Δ (MZ553), and *asf1*Δ (MZ576) cells. The results are expressed relative to the value for the WT strain. *B* and *D*, histone mRNA levels remaining after 30-min HU treatment, calculated as a ratio of mRNA levels in treated (T)/untreated (UT) samples for each individual strain. *A–D*, the experiments were repeated three times, and the results are shown as the means ± SD. Values that are statistically different (*p* < 0.05) from the WT cells are indicated by an *asterisk*. DRC, DNA replication checkpoint; HU, hydroxyurea.

Mutations that compromise the negative feedback regulation of histone transcription, such as *asf1*Δ or *HIR* mutations, render histone genes less responsive to HU-mediated repression (15, 17). To find out if other histone chaperones are required for HU-mediated repression of histone transcription, we determined *HTA1*, *HTA2*, and *HHT2* mRNA levels in WT, *asf1*Δ, *rtt106*Δ, *rtt109*Δ, *cac1*Δ, and *hir1*Δ cells before and after 30 min exposure to HU. The histone mRNA levels in *rtt106*Δ, *rtt109*Δ, *hir1*Δ, and *cac1*Δ cells were comparable or slightly elevated in comparison with WT cells (Fig. 6*A*). The replication stress–induced repression of *HTA1*, *HTA2*, and *HHT2* mRNAs was slightly attenuated in *rtt106*Δ, *rtt109*Δ, *hir1*Δ, and *cac1*Δ cells in comparison with WT cells as shown by the fractions of histone mRNAs remaining after 30 min of HU exposure (Fig. 6*B*). In *asf1*Δ cells, mRNA levels of individual histones were reduced to varying degrees, and the repression of histone genes was significantly attenuated (Fig. 6, *C* and *D*). This is in agreement with the notion that *asf1*Δ mutation renders histone genes immune to HU repression (15). The requirement of Asf1p for HU-mediated repression of histone transcription appears to be independent of Asf1p′s role in promoting H3K56 acetylation because Rtt109p, the histone acetyltransferase responsible for H3K56 acetylation, was not required for HU-mediated repression (Fig. 6*B*). In addition, *hir1*Δ and *rtt106*Δ mutants were competent for HU-mediated repression of histone transcription, indicating that the HIR and Rtt106p chaperones do not have redundant roles with Asf1p in HU-mediated repression of histone genes.

### DRC activation removes PICs and RNA Pol II from histone promoters in Rad53p- and Asf1p-dependent manner

**Figure 7:**
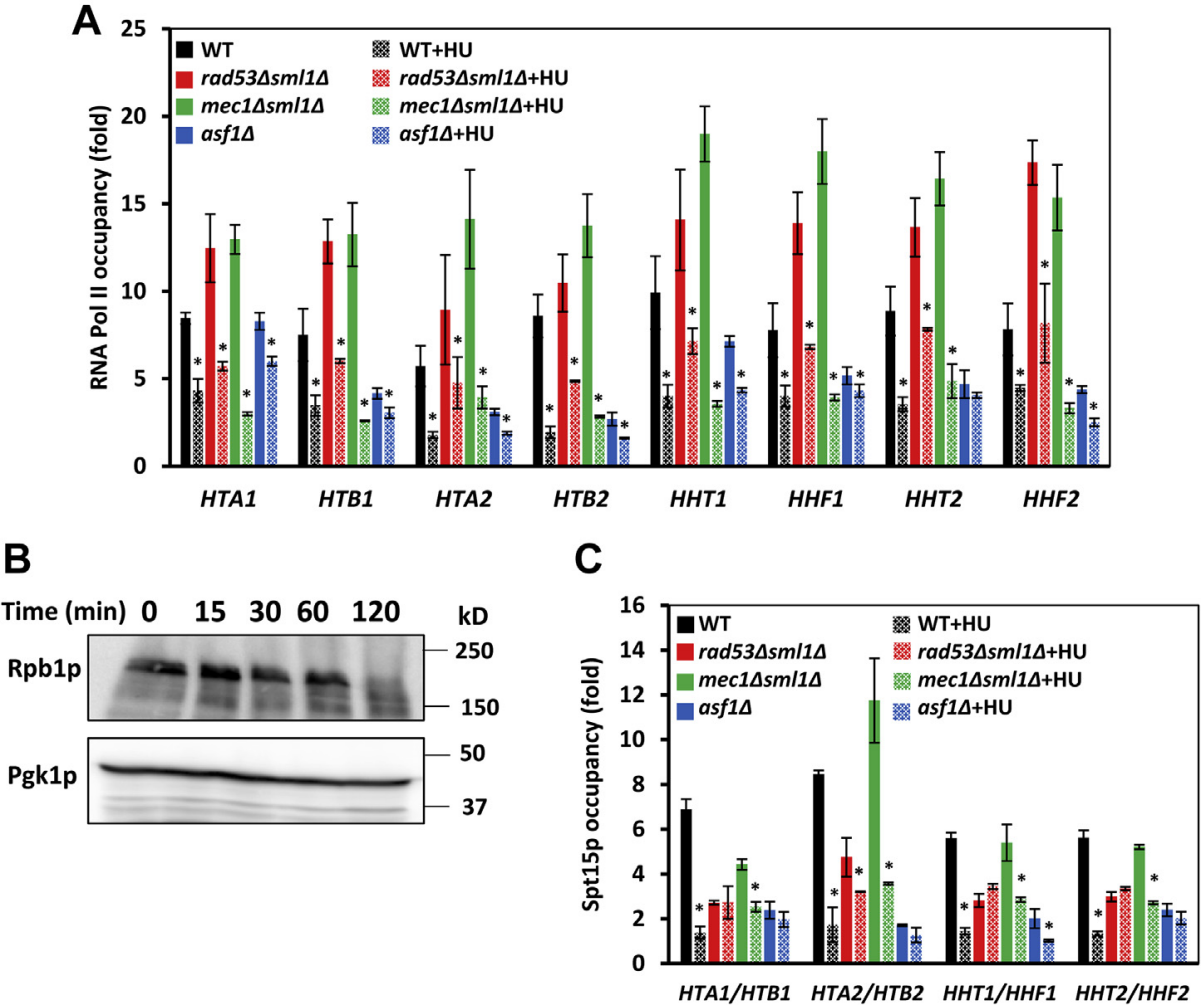
**DRC activation removes RNA Pol II and Spt15p from histone genes.***A* and *C*, occupancies of RNA Pol II and Spt15p at histone genes before and after treatment with 200 mM hydroxyurea (HU) for 15 min in WT, *rad53*Δ*sml1*Δ, *mec1*Δ*sml1*Δ, and *asf1*Δ cells expressing *SPT15* tagged with three copies of the HA epitope (strains AD066, MB159, MB163, and MB191, respectively). Each immunoprecipitation was performed at least three times using different chromatin samples, and the occupancy at the indicated genes was calculated using the *POL1* coding sequence as a negative control. The data are presented as fold occupancy over the *POL1* coding sequence control and represent the means ± SD. Values for the HU-treated samples that are statistically different (*p* < 0.05) from values for the untreated samples in the same strain are indicated by an *asterisk*. *B*, Rpb1p protein levels in WT cells during treatment with 200 mM HU. Western blotting analyses were performed three times, and representative results are shown. Pgk1p served as a loading control. DRC, DNA replication checkpoint.

To determine whether DRC activation affects the assembly of the PICs, we determined the occupancy of Spt15p, the yeast TATA-binding protein, and Rpb1p, the largest subunit of RNA Pol II, at the histone genes in WT, *rad53*Δ*sml1*Δ, *mec1*Δ*sml1*Δ, and *asf1*Δ cells before and after treatment with HU. The occupancies of RNA Pol II at the histone genes were reduced to about 15 to 50% by 15 min HU treatment in the WT cells (Fig. 7*A*). This is in agreement with the reduced histone mRNA levels in HU-treated cells (Fig. 1*A*). The occupancies of RNA Pol II at the histone genes in *rad53*Δ*sml1*Δ cells before HU treatment were somewhat elevated in comparison with WT cells, in agreement with the elevated histone mRNA levels in *rad53*Δ*sml1*Δ cells (Fig. 1*A*). HU treatment reduced the RNA Pol II occupancy at the histone genes in *rad53*Δ*sml1*Δ cells to 27 to 56%, slightly less significantly than in the WT cells. The occupancies of RNA Pol II at the histone genes in *mec1*Δ*sml1*Δ cells before HU treatment were elevated in comparison with the WT cells, and the HU treatment reduced the RNA Pol II occupancy in *mec1*Δ*sml1*Δ cells to 20 to 40%, similarly as in the WT cells. The occupancies of RNA Pol II at the histone genes in *asf1*Δ cells before HU treatment were reduced in comparison with the WT cells (Fig. 7*A*), mirroring reduced histone mRNA levels in *asf1*Δ cells (Fig. 6*C*). In contrast with the WT and *mec1*Δ*sml1*Δ cells, the RNA Pol II occupancies at the histone genes in *asf1*Δ cells were reduced only to about 57 to 83% by the HU treatment (Fig. 7*A*).

Because DNA damage may trigger stalling and arrest of RNA Pol II, leading to polyubiquitination and degradation of Rpb1p, we determined Rpb1p levels during the HU treatment in the WT cells (Fig. 7*B*). The results showed that the 15 min incubation with 200 mM HU, the condition used in our chromatin immunoprecipitation (ChIP) experiments, did not result in degradation of Rpb1p (Fig. 7*B*). This result suggests that the treatment with HU triggers removal of RNA Pol II from the histone genes, but not its degradation.

The occupancies of Spt15p at the histone promoters were similar in the WT and *mec1*Δ*sml1*Δ cells before and after the HU treatment, which reduced Spt15p occupancy to about 20% (Fig. 7*C*). The occupancies of Spt15p in *rad53*Δ*sml1*Δ and *asf1*Δ cells were significantly reduced in comparison with the WT cells; however, the occupancies were not significantly affected by the HU treatment.

### DRC activation evicts Spt10p and Spt21p from histone promoters

**Figure 8:**
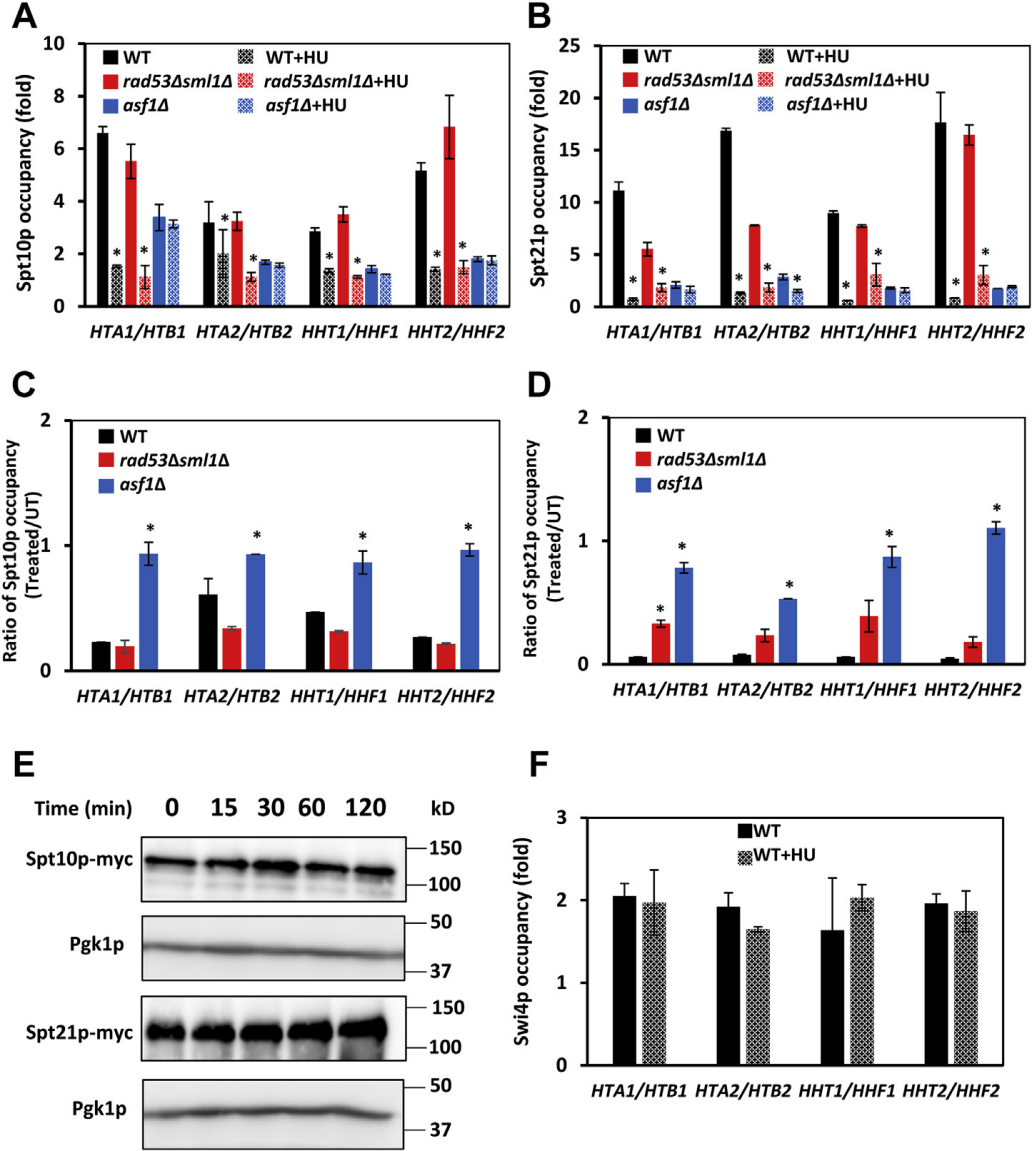
**DRC activation evicts Spt10p and Spt21p from the histone promoters.***A*, occupancy of Spt10p at the histone promoters before and after treatment with 200 mM hydroxyurea (HU) for 15 min in WT, *rad53*Δ*sml1*Δ, and *asf1*Δ cells expressing *SPT10* tagged with myc epitope (strains MB141, MB186, and MB198, respectively). *B*, occupancy of Spt21p at the histone promoters before and after treatment with 200 mM HU for 15 min in WT, *rad53*Δ*sml1*Δ, and *asf1*Δ cells expressing *SPT21* tagged with myc epitope (strains MB150, MB189, and MB195, respectively). Thr ratio of (*C*) Spt10p occupancy and (*D*) Spt21p occupancy at the histone promoters in treated (T)/untreated (UT) samples for WT, *rad53*Δ*sml1*Δ, and *asf1*Δ cells. *E*, Spt10p-myc and Spt21p-myc protein levels in WT cells during treatment with 200 mM HU. Western blotting analyses were performed three times, and representative results are shown. Pgk1p served as a loading control. *F*, occupancy of Swi4p at the histone promoters before and after treatment with 200 mM HU for 15 min in WT cells expressing *SWI4* tagged with the myc epitope (BY4691). Each immunoprecipitation was performed at least three times using different chromatin samples, and the occupancy at the indicated genes was calculated using the *POL1* coding sequence as a negative control. The data are presented as fold occupancy over the *POL1* coding sequence control and represent the means ± SD. Values for the HU-treated samples that are statistically different (*p* < 0.05) from values for the untreated samples in the same strain are indicated by an *asterisk*. DRC, DNA replication checkpoint.

Because Spt10p/Spt21p are the key factors responsible for cell cycle–dependent transcription of histone genes, we determined their occupancy at the histone promoters before and after HU treatment. Similar to Spt15p and RNA Pol II, occupancies of Spt10p and Spt21p at the histone promoters before HU treatment were significantly reduced in *asf1*Δ cells (Fig. 8, *A* and *B*). The requirement of Asf1p for recruitment of Spt10p/Spt21p to histone promoters is not entirely surprising because Asf1p is required for transcriptional activation of several inducible genes by disassembling promoter chromatin (53). These results suggest that Asf1p plays an important role not only in mediating transcriptional response of histones to replication stress imposed by HU but also in the assembly of the PICs at histone promoters in the absence of replication stress. It appears that the recruitment of Sp10p/Spt21p and Asf1p to histone promoters is mutually dependent. The Asf1p occupancy at histone promoters was reduced in *spt10*Δ and *spt21*Δ cells (8), and the occupancy of Spt10p and Spt21p was reduced in *asf1*Δ cells (Fig. 8, *A* and *B*).

HU-mediated replication stress reduced the occupancies of Spt10p and Spt21p in both WT and *rad53*Δ*sml1*Δ cells. However, in *asf1*Δ cells, the Spt10p and Spt21p occupancies were not significantly affected by HU treatment (Fig. 8, *A* and *B*). Cumulatively, these results indicate that the replication stress–induced removal of Spt10p from the histone promoters requires Asf1p but not Rad53p (Fig. 8*A*), as indicated by the fractions of Spt10p remaining at the histone promoters after 15 min HU exposure (Fig. 8*C*). However, the replication stress–induced removal of Spt21p from the histone promoters was attenuated in *rad53*Δ*sml1*Δ cells (Fig. 8*B*) as shown by the fractions of Spt21p remaining at the histone promoters after 15-min HU exposure (Fig. 8*D*). These results suggest that the HU-mediated replication stress inhibits histone transcription by two, perhaps parallel mechanisms. First, Spt10p is removed from the histone promoters in an Asf1p-dependent manner, and second, Rad53p contributes to the removal of Spt21p.

Degradation of Spt10p and Spt21p during incubation with HU was not responsible for the reduced occupancy of both proteins at the histone promoters; while the Spt10p level was unchanged during HU treatment, the Spt21p level slightly increased, probably reflecting accumulation of cells in the S phase (Fig. 8*E*). Cumulatively, these results suggest that the HU-induced replication stress disassembles the PICs and evicts RNA Pol II from the histone genes by a mechanism that depends on Asf1p and Rad53p.

Because the DRC activation–triggered removal of Spt10p and Spt21p from the histone promoters was quite unexpected, we also examined the occupancy of Swi4p, the DNA-binding subunit of the SBF complex, at the histone promoters. Unlike Spt10p and Spt21p, the occupancy of Swi4p at the histone promoters was not affected by HU treatment (Fig. 8*F*). This finding indicates that the removal of Spt10p and Spt21p from the histone promoters is not a common response of transcription factors to DRC activation.

## Discussion

In mammalian cells, replication-dependent histone mRNAs are not polyadenylated, but their sequence in the 3′ untranslated region forms a stem loop structure bound by a stem loop–binding protein. At the end of the S phase, or when the DNA replication is inhibited during the S phase, histone mRNAs are degraded in a stem loop–dependent manner by 5′ to 3′ and 3′ to 5′ pathways (54, 55). Unlike in mammalian cells, histone mRNAs in *S. cerevisiae* are polyadenylated. Early studies in budding yeast showed that when DNA replication during the S phase is inhibited using temperature-sensitive *cdc8* mutation, histone *HTA1* and *HTB1* mRNAs disappear faster than during the normal end of the S phase (20). These results were interpreted that inhibition of DNA synthesis results in decay of histone mRNAs (20). Subsequent studies utilized fusion of histone *HTA1* gene with *lacZ* (21). When this fusion was expressed from histone promoter, it behaved identically to the endogenous histones, and the corresponding *HTA1-lacZ* mRNA was repressed when DNA synthesis was inhibited using the *cdc8* mutation or HU. However, when this fusion was expressed from the *GAL10* promoter, the fusion mRNA was insensitive to HU, suggesting that the coupling between DNA synthesis and histone mRNAs is due to transcriptional regulation. Nevertheless, when histone genes containing full coding and 3′ end noncoding sequences were expressed from a constitutive promoter, the corresponding mRNAs still accumulated during the S phase, albeit to a lower level than endogenous histone mRNAs. This result suggested that part of the coding and 3′ end noncoding sequences are required for post-transcriptional regulation and that histone mRNAs are more stable during the S phase (21). Interestingly, the accumulation of *HTB1* mRNA expressed from this constitutive promoter during the S phase was not significantly affected by HU, suggesting that DRC likely does not affect decay of histone mRNAs (21). However, because these studies (21) did not use thiolutin or another inhibitor of RNA synthesis, the results probably reflect contributions of both synthesis and decay of histone mRNAs. The role of 3′ end sequence of *HTB1* gene in cell cycle regulation was confirmed with a fusion construct that contained the *GAL1* promoter and bacterial neomycin phosphotransferase II gene (neo) fused to a small portion of the 3′ end coding sequence and the entire noncoding sequence of histone *HTB1* (22, 23). This *neo-HTB1* message accumulated during the S phase of the undisturbed cell cycle; however, these studies did not address whether DRC regulates stability of this heterologous mRNA (22, 23). Subsequent studies showed that histone mRNAs are decayed by both 5′ to 3′ and 3′ to 5′ pathways. Inactivation of *RRP6*, component of the nuclear exosome, or *TRF4/TRF5*, components of the TRAMP complex, elevates histone mRNA levels (24, 25). TRAMP complex polyadenylates RNA substrates before their degradation by the nuclear exosome. Histone mRNA levels are also elevated in the *lsm1*Δ mutant (26, 56); *LSM1* is a component of the Lsm1-7–Pat1 complex that stimulates decapping of mRNA. These results are consistent with our data (Fig. 3*B*) that indicate that histone mRNAs are regulated by degradation in 5′ to 3′ and 3′ to 5′ decay pathways. Inactivation of *PAN2*, *CCR4*, *DCP2*, *XRN1*, and *SKI2*, major players in both 5′ to 3′ and 3′ to 5′ decay pathways, resulted in significantly increased half-lives of histone mRNAs (Fig. 3*B*).

Histone mRNA levels correlate with DNA replication and must be suppressed when DNA replication is inhibited to prevent toxicity and genome instability due to free nonchromatinized histone proteins. One could argue that because half-lives of histone mRNAs in yeast are quite short under normal conditions (Fig. 3*B*) (39, 40, 41), further destabilization by DRC activation would not provide a sufficiently effective regulatory strategy. Our data appear to support this logic and show that activation of DDR/DRC does not destabilize histone mRNAs, but inhibits transcription of histone genes, as indicated by the disassembly of the PICs at the histone promoters after HU treatment (Figs. 7 and 8).

The three simplest and mutually not-exclusive mechanisms that explain the disassembly of the PICs and removal of RNA Pol II from the histone promoters during DNA replication stress are (i) active disassembly driven by direct phosphorylation by one of the DRC kinases, (ii) negative feedback regulation driven by free histone proteins that accumulate when DNA replication stops or slows down, or (iii) degradation of RNA Poll triggered by DNA replication stress.

We believe that a combination of the first two possibilities is likely. The removal of RNA Pol II, Spt15p, and Spt21p after HU treatment was partly attenuated in *rad53*Δ*sml1*Δ cells, indicating the involvement of Rad53p. Rad53p phosphorylates several transcription factors that may account for the role of DRC in the assembly and disassembly of the PICs at the histone promoters. Rad53p directly phosphorylates Mbp1p, Swi4p, and Swi6p (47, 48, 51, 57), activates transcription of MBF targets by phosphorylating MBF repressor Nrm1p (49, 50), and downregulates Swi6p and transcription of SBF targets (47, 48). However, our data show that the transcription of histone genes in *swi6*Δ cells during the S phase is repressed by HU (Fig. 5), indicating that SBF and MBF complexes are not responsible or at least not solely responsible for the DRC-mediated repression of histone genes. Another Rad53p target is Spt21p. Rad53p phosphorylates Spt21p in the absence of DRC activation, and this phosphorylation reduces histone gene transcription (37). This negative role of Rad53p on the transcription of histone genes in the absence of DRC activation is consistent with our data showing elevated levels of histone mRNAs and elevated occupancy of RNA Pol II at the histone genes in *rad53*Δ*sml1*Δ cells (Figs. 2*A* and 7*A*). However, because the removal of Spt21p from the histone promoters after HU treatment was only partly dependent on Rad53p (Fig. 8, *B* and *D*), it is not clear whether this phosphorylation is responsible for the removal of Spt21p from the histone promoters after DRC activation. Interestingly, proteome-wide screens identified Spt21p as a target of DNA damage–induced phosphorylation (57), and a genome-wide study of changes in protein localization found that the localization pattern of Spt21p changes from nuclear foci to diffuse nuclear signal during DNA replication stress (58).

The second possible mechanism for the disassembly of the PICs at the histone promoters during DNA replication stress is negative feedback regulation driven by free histone proteins that accumulate when DNA replication slows down. The model posits that the HIR complex binds to NEG regions and recruits Asf1p to histone promoters. During replication stress or at the end of the S phase, free histones bind to Rtt106p. The histone–Rtt106p complex then binds to Asf1p at the histone promoters, causing transcriptional repression (17, 18). The recruitment of Asf1p to histone promoters requires Spt10p and Spt21p (8) and does not depend entirely on the HIR complex (17).

Our data suggest that Asf1p is required for HU-mediated transcriptional repression of histone genes and that the HIR complex and Rtt106p are redundant in this process (Fig. 5). The simplest although rather hypothetical interpretation of our results is that under conditions of HU-mediated replication stress, the free histones bind directly to Asf1p, which is recruited to the histone promoters by Spt10p/Spt21p. It is tempting to speculate that the recruitment of histone-charged Asf1p leads to dissociation of Spt10p/Spt21p from the histone promoters, perhaps with the assistance of Rad53p. We need to point out, however, that eviction of a transcriptional factor by a histone chaperone charged with histone has not been described yet. Replication stress or the end of the S phase is not the only situation that generates free nonchromatinized histones. During transcription, histones are evicted from chromatin in front and reassembled behind elongating RNA Pol II (59, 60, 61). The redeposition of histones in the wake of RNA Pol II is mediated by histone chaperones FACT and Spt6p. However, the redeposition of histones evicted from transcribed chromatin does not appear to result in eviction of transcription factors. In the absence of FACT, evicted histones repress *CLN3* transcription without affecting the occupancy of the transcription factor Mcm1p at the *CLN3* promoter (60).

The third possible mechanism for the removal of RNA Pol II from the histone promoters during DNA replication stress is degradation of Rpb1p, the largest subunit of RNA Pol II. Rpb1p polyubiquitination and degradation occur when elongating RNA Pol II arrests and cannot be restarted as a “mechanism of last resort” to make DNA accessible for DNA repair and/or continued transcription (62). Conditions leading to RNA Pol II stalling and/or arrest include DNA damage and various forms of transcription stress (62, 63, 64, 65). RNA Pol II is also evicted from chromatin and degraded after HU-induced replication stress (66). While we cannot completely eliminate contribution of Rpb1p degradation to HU-induced transcriptional inhibition of histone genes, we believe that it does not represent the main mechanism for the following reasons. First, we have not detected Rpb1p degradation after 15 min HU treatment (Fig. 7*B*), while the occupancy of Rpb1p at the histone genes decreased to about 30 to 50%. Second, HU treatment removes Spt10p, Spt21p, and Spt15p from the histone promoters, thus disassembling the PIC and preventing recruitment of RNA Pol II to histone promoters, making Rpb1p degradation unnecessary for regulation of histone gene transcription.

Overall, our data suggest that the mechanism of repression of histone genes by HU requires Asf1p and is mediated by reduced occupancy of Spt10p and Spt21p that triggers disassembly of the PICs at the histone promoters. However, the mechanism might be more complex, given that Asf1p is found in complex with Rad53p and the two proteins dissociate in response to replication stress (67, 68, 69).

## Experimental procedures

### Yeast strains and media

All yeast strains are listed in Table 1. Standard genetic techniques were used to manipulate yeast strains and to introduce mutations from non-W303 strains into the W303 background (79). Cells were grown in the YPD medium (1% yeast extract, 2% Bacto Peptone, 2% glucose), YEP medium (1% yeast extract, 2% Bacto peptone) containing 0.05% glucose, or under selection in synthetic complete medium containing 2% glucose and, when appropriate, lacking specific nutrients to select for a strain with a particular genotype. Cell-cycle arrest in the G1 phase by α-factor was carried out as described (29).

**Table 1:** Yeast strains used in this study

Strain	Genotype	Source/reference
W303-1a	*MATa ade2-1 his3-11,15 leu2-3,112 trp1-1 ura3-1 ssd1-d2 can1-100*	R. Rothstein
W303-1α	*MAT*α *ade2-1 his3-11,15 leu2-3,112 trp1-1 ura3-1 ssd1-d2 can1-100*	R. Rothstein
W303	*MATa/MAT*α *ade2-1/ade2-1 his3-11,15/his3-11,15 leu2-3,112/leu2-3,112 trp1-1/trp1-1ura3-1/ura3-1 can1-100/can1-100*	R. Rothstein
PB119	*MATa ade2-1 his3-11,15 leu2-3,112 trp1-1 ura3-1 ssd1-d2 can1-100 dun1::KAN*	(29)
SN159	*MATa ade2-1 his3-11,15 leu2-3,112 trp1-1 ura3-1 ssd1-d2 can1-100 tel1::HIS3*	(29)
SN136	*MATα ade2-1 his3-11,15 leu2-3,112 trp1-1 ura3-1 ssd1-d2 can1-100 chk1::HIS3*	(29)
SN117	*MATa ade2-1 his3-11,15 leu2-3,112 trp1-1 ura3-1 ssd1-d2 can1-100 mec1::HIS3 sml1::KAN*	(29)
LG606	*MATa ade2-1 his3-11,15 leu2-3,112 trp1-1 ura3-1 ssd1-d2 can1-100 rad53::KAN sml1::HYG*	(29)
LG731	*MATa ade2-1 his3-11,15 leu2-3,112 trp1-1 ura3-1 ssd1-d2 can1-100 rad52::TRP1*	(29)
DY5780	*MATa ade2-1 his3-11,15 leu2-3,112 trp1-1 ura3-1 ssd1-d2 can1-100 swi6::TRP1*	(70)
FY2195	*MATa his3200 leu20 ura30 lys2-128 δ trp163 SPT10-MYC::kanMX*	(11)
MB141	*MATa ade2-1 his3-11,15 leu2-3,112 trp1-1 ura3-1 ssd1-d2 can1-100 SPT10-MYC::kanMX*	This study
FY2194	*MATa his3200 leu20 ura30 lys2-128δ trp163 SPT21-MYC::kanMX*	(11)
MB150	*MATa ade2-1 his3-11,15 leu2-3,112 trp1-1 ura3-1 ssd1-d2 can1-100 SPT21-MYC::kanMX*	This study
AD066	*MATα ade2-1 his3-11,15 leu2-3,112 trp1-1 ura3-1 ssd1-d2 can1-100 SPT15-3HA::URA3*	(71)
yRP1619	*MATa trp1 ura3-52 leu2-3, 112 cup1D::LEU2PM his4-539 pan2::URA3*	(72)
MB123	*MATa ade2-1 his3-11,15 leu2-3,112 trp1-1 ura3-1 ssd1-d2 can1-100 pan2::URA3*	This study
DY3462-4	*MATa his4-912d his4-912d-ADE2 lys2-128d can1 leu2 ura3 ccr4::URA3*	(73)
SM096	*MATa ade2-1 his3-11,15 leu2-3,112 trp1-1 ura3-1 ssd1-d2 can1-100 ccr4::URA3*	This study
yRP2859	*MATa ade2-1 his3-11,15 leu2-3,112 trp1-1 ura3-1 can1-100 dcp2::HIS3*	(74)
yRP1199	*MATα his4-539 leu2-3,112 trp1-Δ1 ura3-52 xrn1::URA3*	(75)
MB115	*MATa ade2-1 his3-11,15 leu2-3,112 trp1-1 ura3-1 ssd1-d2 can1-100 xrn1::URA3*	This study
yRP1192	*MATα his4-539 leu2-3,112 lys2-201 trp1-Δ1 ura3-52 ski2::LEU2*	(75)
MB133	*MATa ade2-1 his3-11,15 leu2-3,112 trp1-1 ura3-1 ssd1-d2 can1-100 ski2::LEU2*	This study
yRP1193	*MATα his4-539 leu2-3,112 lys2-201 trp1-Δ1 ura3-52 ski3::TRP1*	(75)
MB109	*MATa ade2-1 his3-11,15 leu2-3,112 trp1-1 ura3-1 ssd1-d2 can1-100 ski3::TRP1*	This study
yRP1377	*MATα leu2-3 112 trp1 ura3-52 cup1::LEU2/PGK1pG/MFA2pG rrp6::URA3*	(76)
MB120	*MATa ade2-1 his3-11,15 leu2-3,112 trp1-1 ura3-1 ssd1-d2 can1-100 rrp6::URA3*	This study
MB159	*MATa ade2-1 his3-11*,*15 leu2-3,112 trp1-1 ura3-1 ssd1-d2 can1-100 rad53::KAN sml1::HYG SPT15-3HA::URA3*	This study
MB186	*MATa ade2-1 his3-11*,*15 leu2-3,112 trp1-1 ura3-1 ssd1-d2 can1-100 rad53::HIS3 sml1::HYG SPT10-MYC::kanMX*	This study
MB189	*MATa ade2-1 his3-11*,*15 leu2-3,112 trp1-1 ura3-1 ssd1-d2 can1-100 rad53::HIS3 sml1::HYG SPT21MYC::kanMX*	This study
MB163	*MATa ade2-1 his3-11*,*15 leu2-3*,*112 trp1-1 ura3-1 ssd1-d2 can1-100 mec1::HIS3 sml1::KAN SPT15-3HA::URA3*	This study
MB191	*MATa ade2-1 his3-11*,*15 leu2-3,112 trp1-1 ura3-1 ssd1-d2 can1-100 asf1*::*HIS3 SPT15-3HA::URA3*	This study
MB195	*MATa ade2-1 his3-11*,*15 leu2-3,112 trp1-1 ura3-1 ssd1-d2 can1-100 asf1*::*HIS3 SPT21MYC::kanMX*	This study
MB198	*MATa ade2-1 his3-11*,*15 leu2-3,112 trp1-1 ura3-1 ssd1-d2 can1-100 asf1*::*HIS3 SPT10-MYC::kanMX*	This study
BY4691	*MATa his3Δ1 leu2Δ0 met15Δ0 ura3Δ0 SWI4-myc::URA3*	(77)
MZ576	*MATa ade2-1 his3-11*,*15 leu2-3,112 trp1-1 ura3-1 ssd1-d2 can1-100 asf1*::*HIS3*	(78)
MZ642	*MATa ade2-1 his3-11*,*15 leu2-3,112 trp1-1 ura3-1 ssd1-d2 can1-100 rtt106*::*KAN*	(78)
MZ655	*MATa ade2-1 his3-11*,*15 leu2-3,112 trp1-1 ura3-1 ssd1-d2 can1-100 rtt109*::*KAN*	(78)
MZ553	*MATa ade2-1 his3-11*,*15 leu2-3*,*112 trp1-1 ura3-1 ssd1-d2 can1-100 cac1*::*LEU2*	(78)
MZ700	*MATa ade2-1 his3-11*,*15 leu2-3*,*112 trp1-1 ura3-1 ssd1-d2 can1-100 hir1*::*HIS3*	(78)
MB181	*MATa ade2-1 his3-11*,*15 leu2-3*,*112 trp1-1 ura3-1 ssd1-d2 can1-100 mec1::TRP1 tel1::HIS3 [2μ-LEU2-RNR1]*	This study

### Western blotting

Yeast cells were inoculated to an *A*_600_ = 0.1 and grown in the YPD medium to an *A*_600_ = 1.0. Four *A*_600_ units were harvested and immediately boiled in the SDS sample buffer. Denatured proteins were separated on a denaturing polyacrylamide gel, and Western blotting with anti-histone H3 polyclonal antibody (ab1791; Abcam) at a dilution of 1:1000, anti-RNA Polymerase II Rpb1p monoclonal antibody (8WG16; 664912, BioLegend) at a dilution of 1:500, anti-myc monoclonal antibody (9B11; 2276S; Cell Signaling Technology) at a dilution of 1:1000, and anti-Pgk1p monoclonal antibody (22C5D8; 459250; Invitrogen) at a dilution of 1:3000 was carried out as described previously (29).

### RT-qPCR

The procedures to extract total RNA from yeast cells and perform RT-qPCR were as previously described (29, 80). The primers used are as follows:

*ACT1* (5′-TATGTGTAAAGCCGGTTTTGC-3′ and 5′-GACAATAC CGTGTTCAATTGGG-3′), *RDN25* (5′-GGAATGTAGCTTGCCTCGGT-3′ and 5′-TTACGTCGCAGTCCTCAGTC-3′), *SIC1* (5′-GCTTACGTCTCCTCAACGCT-3′ and 5′-CACATTTTGCTGCGTGGGAA-3′), *SWI5* (5′-CTAGAGGCAGTGACCCTTCG-3′ and 5′-ATCAGAAAGTGGTCCCAGCG-3′), *RNR1* (5′- ACCGCTCGTATATCACGCTTAT-3′ and 5′- TTGTGACACCTTCATAGACACCA-3′), *HTA1* (5′-ACGTTACCATTGCCCAAGGT-3′ and 5′-GTTTAGTTCCTTCCGCCTTCTT-3′), *HTA2* (5′-GCTATTGGGTAATGTTACCATCG-3′ and 5′-TGCTTTGTTTCTTTTCAACTCAGT-3′), *HTB1* (5′-TGGCTGCGTATAACAAGAAGTCT-3′ and 5′-CCAAAGGAAGTGATTTCATTATGC-3′), *HTB2* (5′-TGCTCTATACTCAAACCAACAACA-3′ and 5′-ATCTCTTCTTACCATCGACGGA-3′), *HHT1* (5′-AATATATAAACGCAAACAATGGC-3′ and 5′-GATTTTCTGGCAGCCTTAGAAG-3′), *HHT2* (5′-TTGAAGACACTAATCTGGCTGCT-3′ and 5′-GATGTCCCCCCAGTCTAAATG-3′), *HHF1* (5′-GAATCCGTCATCAGAGACTCTGTT-3′ and 5′-TGCTTGTTGTTACCGTTTTCTTAG-3′), and *HHF2* (5′-TCTGTTACTTACACTGAACACGCC-3′ and 5′-AAACACCGATTGTTTAACCACC-3′). Primers *HTA1*, *HTA2*, *HTB1*, *HTB2*, *HHT1*, *HHT2*, *HHF1*, and *HHF2* recognize individual histone mRNAs and amplify the 3′ ends of the translated sequences and adjacent not-translated sequences. Primers *HTA1*/*HTA2*, *HTB1*/*HTB2*, *HHT1*/*HHT2*, and *HHF1/HHF2* were designed so that they measure expression of both genes for that particular histone (*HTA1* and *HTA2*, *HTB1* and *HTB2*, *HHT1* and *HHT2*, and *HHF1* and *HHF2*): *HTA1*/2 (5′-CGGTGGTAAAGGTGGTAAAGC-3′ and 5′-TGGAGCACC AGAACCAATTC-3′), *HTB1/2* (5′-CAAAGTTTTGAAGCAAACTCACCC-3′ and 5′-GCCAATTTAGAAGCTTCAGTAGC-3′). *HHT1/2* (5′-GAAGCCTCACAGATATAAGCCAG-3′ and 5′-ATCTTGAGCG ATTTCTCTGACC-3′), *HHF1/2* (5′-CCAAGCGTCACAGAAAGATTCTA -3′ and 5′-ACCAGAAATACGCTTG ACACCA-3′).

### Histone mRNA decay rates

The half-lives (t_1/2_) of histone mRNAs were determined using transcriptional shutoff with thiolutin as described (81, 82). Yeast cells were inoculated to an *A*_600_ = 0.1 and grown in the YPD medium to an *A*_600_ = 0.8. Thiolutin was added to 8 μg/ml, and culture samples were removed during 0 to 60 min incubation. Total RNA was isolated as described above, and histone mRNA levels were determined by RT-qPCR using *RDN25* as a control. The half-lives of individual histone mRNAs were determined in Microsoft Excel from logarithmic plots of each remaining mRNA at different times after the transcriptional shutoff.

### ChIP assays

*In vivo* chromatin crosslinking and immunoprecipitation was performed essentially as described (80). For RNA Pol II and Spt15p-HA ChIP, the cells were crosslinked for 15 min. For Spt10p-myc and Spt21p-myc ChIP, the cells were crosslinked for 45 min. Immunoprecipitation was performed with the following antibodies: anti-RNA Polymerase II Rpb1p monoclonal antibody (8WG16; 664912, BioLegend), anti-myc monoclonal antibody (9B11; 2276S; Cell Signaling), and anti-HA monoclonal antibody (F-7; sc-7392X; Santa Cruz Biotechnology). The primers used for qPCR are as follows: *POL1* (5′-TCCTGACAAAGA AGGCAATAGAAG-3′ and 5′-TAAAACACCCTGATCCACCTCTG-3′), *HTA1/HTB1* promoter (5′-ATAGTTAACGACCCAACCGC-3′ and 5′-CTCCATTCCAATAGCTTCGCA-3′), *HTA2/HTB2* promoter (5′-CGTCGCGTTTATGGCCCC-3′ and 5′-GAGAACACCGCTTTATTAGGC-3′), *HHT1/HHF1* promoter (5′-GAACGCGGTTTCCAAATTCG-3′ and 5′-GCAGAGCAAGGAAATGTGAGA-3′), and *HHT2/HHF2* promoter (5′-GCCAATAGTTTCACGCGCTT-3′ and 5′-ACGTCCTGCCATACAAATGC-3′). Primers that recognize individual histone genes were used for RNA Pol II ChIP.

### Flow cytometry

The DNA contents of cells were determined by flow cytometry of SYTOX Green–stained cells using the MilliporeSigma Guava easyCyte flow cytometer as described (83).

### Statistical analysis

The results represent at least three independent experiments. Numerical results are presented as the means ± SD. Data were analyzed by using an InStat software package (GraphPad). Statistical significance was evaluated by one-way ANOVA, and *p* < 0.05 was considered significant.

## Data availability

All data are included within the article.

## Conflict of interest

The authors declare that they have no conflict of interest with the contents of this article.

## References

[1] Eriksson P.R., Ganguli D., Nagarajavel V., Clark D.J. (2012). Regulation of histone gene expression in budding yeast. Genetics.

[2] Kurat C.F., Recht J., Radovani E., Durbic T., Andrews B., Fillingham J. (2013). Regulation of histone gene transcription in yeast. Cell. Mol. Life Sci..

[3] Gasch A.P., Huang M., Metzner S., Botstein D., Elledge S.J., Brown P.O. (2001). Genomic expression responses to DNA-damaging agents and the regulatory role of the yeast ATR homolog Mec1p. Mol. Biol. Cell.

[4] Su C., Gao G., Schneider S., Helt C., Weiss C., O'Reilly M.A., Bohmann D., Zhao J. (2004). DNA damage induces downregulation of histone gene expression through the G_1_ checkpoint pathway. EMBO J..

[5] Libuda D.E., Winston F. (2010). Alterations in DNA replication and histone levels promote histone gene amplification in Saccharomyces cerevisiae. Genetics.

[6] Osley M.A., Gould J., Kim S., Kane M., Hereford L. (1986). Identification of sequences in a yeast histone promoter involved in periodic transcription. Cell.

[7] Eriksson P.R., Mendiratta G., McLaughlin N.B., Wolfsberg T.G., Marino-Ramirez L., Pompa T.A., Jainerin M., Landsman D., Shen C.H., Clark D.J. (2005). Global regulation by the yeast Spt10 protein is mediated through chromatin structure and the histone upstream activating sequence elements. Mol. Cell. Biol..

[8] Kurat C.F., Lambert J.-P., Petschnigg J., Friesen H., Pawson T., Rosebrock A., Gingras A.-C., Fillingham J., Andrews B. (2014). Cell cycle-regulated oscillator coordinates core histone gene transcription through histone acetylation. Proc. Natl. Acad. Sci. U. S. A..

[9] Mendiratta G., Eriksson P.R., Shen C.H., Clark D.J. (2006). The DNA-binding domain of the yeast Spt10p activator includes a zinc finger that is homologous to foamy virus integrase. J. Biol. Chem..

[10] Mendiratta G., Eriksson P.R., Clark D.J. (2007). Cooperative binding of the yeast Spt10p activator to the histone upstream activating sequences is mediated through an N-terminal dimerization domain. Nucleic Acids Res..

[11] Hess D., Liu B., Roan N.R., Sternglanz R., Winston F. (2004). Spt10-dependent transcriptional activation in Saccharomyces cerevisiae requires both the Spt10 acetyltransferase domain and Spt21. Mol. Cell. Biol..

[12] Hess D., Winston F. (2005). Evidence that Spt10 and Spt21 of Saccharomyces cerevisiae play distinct roles *in vivo* and functionally interact with MCB-binding factor, SCB-binding factor and Snf1. Genetics.

[13] Dimova D., Nackerdien Z., Furgeson S., Eguchi S., Osley M.A. (1999). A role for transcriptional repressors in targeting the yeast Swi/Snf complex. Mol. Cell..

[14] Eriksson P.R., Ganguli D., Clark D.J. (2011). Spt10 and Swi4 control the timing of histone H2A/H2B gene activation in budding yeast. Mol. Cell. Biol..

[15] Sutton A., Bucaria J., Osley M.A., Sternglanz R. (2001). Yeast ASF1 protein is required for cell cycle regulation of histone gene transcription. Genetics.

[16] Fillingham J., Kainth P., Lambert J.P., van Bakel H., Tsui K., Peña-Castillo L., Nislow C., Figeys D., Hughes T.R., Greenblatt J., Andrews B.J. (2009). Two-color cell array screen reveals interdependent roles for histone chaperones and a chromatin boundary regulator in histone gene repression. Mol. Cell.

[17] Zunder R.M., Rine J. (2012). Direct interplay among histones, histone chaperones, and a chromatin boundary protein in the control of histone gene expression. Mol. Cell. Biol..

[18] Silva A.C., Xu X., Kim H.-S., Fillingham J., Kislinger T., Mennella T.A., Keogh M.-C. (2012). The replication-independent histone H3-H4 chaperones HIR, ASF1, and RTT106 co-operate to maintain promoter fidelity. J*.* Biol. Chem..

[19] Parker R. (2012). RNA degradation in Saccharomyces cerevisiae. Genetics.

[20] Hereford L.M., Osley M.A., Ludwig J.R., McLaughlin C.S. (1981). Cell-cycle regulation of yeast histone mRNA. Cell.

[21] Lycan D.E., Osley M.A., Hereford L.M. (1987). Role of transcriptional and posttranscriptional regulation in expression of histone genes in Saccharomyces cerevisiae. Mol. Cell. Biol..

[22] Xu H.X., Johnson L., Grunstein M. (1990). Coding and noncoding sequences at the 3' end of yeast histone H2B mRNA confer cell cycle regulation. Mol. Cell. Biol..

[23] Campbell S.G., Li Del Olmo M., Beglan P., Bond U. (2002). A sequence element downstream of the yeast HTB1 gene contributes to mRNA 3' processing and cell cycle regulation. Mol. Cell. Biol..

[24] Canavan R., Bond U. (2007). Deletion of the nuclear exosome component RRP6 leads to continued accumulation of the histone mRNA HTB1 in S-phase of the cell cycle in Saccharomyces cerevisiae. Nucleic Acids Res..

[25] Reis C.C., Campbell J.L. (2007). Contribution of Trf4/5 and the nuclear exosome to genome stability through regulation of histone mRNA levels in Saccharomyces cerevisiae. Genetics.

[26] Herrero A.B., Moreno S. (2011). Lsm1 promotes genomic stability by controlling histone mRNA decay. EMBO J..

[27] Bolzan A.D., Bianchi M.S. (2018). DNA and chromosome damage induced by bleomycin in mammalian cells: An update. Mutat. Res..

[28] Friedberg E.C. (1995). Out of the shadows and into the light: The emergence of DNA repair. Trends Biochem. Sci..

[29] Bu P., Nagar S., Bhagwat M., Kaur P., Shah A., Zeng J., Vancurova I., Vancura A. (2019). DNA damage response activates respiration and thereby enlarges dNTP pools to promote cell survival in budding yeast. J. Biol. Chem..

[30] Mortensen U.H., Lisby M., Rothstein R. (2009). Rad52. Curr. Biol..

[31] Alvino G.M., Collingwood D., Murphy J.M., Delrow J., Brewer B.J., Raghuraman M.K. (2007). Replication in hydroxyurea: it's a matter of time. Mol. Cell Biol..

[32] Matmati N., Kitagaki H., Montefusco D., Mohanty B.K., Hannun Y.A. (2009). Hydroxyurea sensitivity reveals a role for ISC1 in the regulation of G2/M. J. Biol. Chem..

[33] Koch C., Nasmyth K. (1994). Cell cycle regulated transcription in yeast. Curr. Opin. Cell Biol..

[34] Cho R.J., Campbell M.J., Winzeler E.A., Steinmetz L., Conway A., Wodicka L., Wolfsberg T.G., Gabrielian A.E., Landsman D., Lockhart D.J., Davis R.W. (1998). A genome-wide transcriptional analysis of the mitotic cell cycle. Mol. Cell.

[35] Simon I., Barnett J., Hannett N., Harbison C.T., Rinaldi N.J., Volkert T.L., Wyrick J.J., Zeitlinger J., Gifford D.K., Jaakkola T.S., Young R.A. (2001). Serial regulation of transcriptional regulators in the yeast cell cycle. Cell.

[36] Zhao X., Chabes A., Domkin V., Thelander L., Rothstein R. (2001). The ribonucleotide reductase inhibitor Sml1 is a new target of the Mec1/Rad53 kinase cascade during growth and in response to DNA damage. EMBO J..

[37] Bruhn C., Ajazi A., Ferrari E., Lanz M.C., Batrin R., Choudhary R., Walvekar A., Laxman S., Longhese M.P., Fabre E., Smolka M.B., Foiani M. (2020). The Rad53CHK1/CHK2-Spt21NPAT and Tel1ATM axes couple glucose tolerance to histone dosage and subtelomeric silencing. Nat. Commun..

[38] Lao J.P., Ulrich K.M., Johnson J.R., Newton B.W., Vashisht A.A., Wohlschlegel J.A., Krogan N.J., Toczyski D.P. (2018). The yeast DNA damage checkpoint kinase Rad53 targets the exoribonuclease, Xrn1. G3.

[39] Wang Y., Liu C.L., Storey J.D., Tibshirani R.J., Herschlag D., Brown P.O. (2002). Precision and functional specificity in mRNA decay. Proc. Natl. Acad. Sci. U. S. A..

[40] Munchel S.E., Shultzaberger R.K., Takizawa N., Weis K. (2011). Dynamic profiling of mRNA turnover reveals gene-specific and system-wide regulation of mRNA decay. Mol. Biol. Cell.

[41] Geisberg J.V., Moqtaderi Z., Fan X., Ozsolak F., Struhl K. (2014). Global analysis of mRNA isoform half-lives reveals stabilizing and destabilizing elements in yeast. Cell.

[42] Haimovich G., Medina D.A., Causse S.Z., Garber M., Millán-Zambrano G., Barkai O., Chávez S., Pérez-Ortín J.E., Darzacq X., Choder M. (2013). Gene expression is circular: Factors for mRNA degradation also foster mRNA synthesis. Cell.

[43] Sun M., Schwalb B., Pirkl N., Maier K.C., Schenk A., Failmezger H., Tresch A., Cramer P. (2013). Global analysis of eukaryotic mRNA degradation reveals Xrn1-dependent buffering of transcript levels. Mol. Cell.

[44] Braun K.A., Young E.T. (2014). Coupling mRNA synthesis and decay. Mol. Cell. Biol..

[45] Timmers H.T.M., Tora L. (2018). Transcript buffering: A balancing Act between mRNA synthesis and mRNA degradation. Mol. Cell.

[46] Buchan J.R., Parker R. (2009). Eukaryotic stress granules: The ins and outs of translation. Mol. Cell..

[47] Sidorova J.M., Breeden L.L. (1997). Rad53-dependent phosphorylation of Swi6 and down-regulation of *CLN1* and *CLN2* transcription occur in response to DNA damage in *Saccharomyces cerevisiae*. Genes Dev..

[48] Sidorova J.M., Breeden L.L. (2003). Rad53 checkpoint kinase phosphorylation site preference identified in the Swi6 protein of *Saccharomyces cerevisiae*. Mol. Cell. Biol..

[49] Travesa A., Kuo D., de Bruin R.A.M., Kalashnikova T.I., Guaderrama M., Thai K., Aslanian A., Smolka M.B., Yates J.R., Ideker T., Wittenberg C. (2012). DNA replication stress differentially regulates G1/S genes via Rad53-dependent inactivation of Nrm1. EMBO J..

[50] Bastos de Oliveira F.M., Harris M.R., Brazauskas P., de Bruin R.A.M., Smolka M.B. (2012). Linking DNA replication checkpoint to MBF cell-cycle transcription reveals a distinct class of G1/S genes. EMBO J..

[51] Jaehnig E.J., Kuo D., Hombauer H., Ideker T.G., Kolodner R.D. (2013). Checkpoint kinases regulate a global network of transcription factors in response to DNA damage. Cell Rep..

[52] Dirick L., Moll T., Auer H., Nasmyth K. (1992). A central role for SWI6 in modulating cell cycle Start-specific transcription in yeast. Nature.

[53] Adkins M.W., Williams S.K., Linger J., Tyler J.K. (2007). Chromatin disassembly from the PHO5 promoter is essential for the recruitment of the general transcription machinery and coactivators. Mol. Cell. Biol..

[54] Marzluff W.F., Wagner E.J., Duronio R.J. (2008). Metabolism and regulation of canonical histone mRNAs: Life without a poly(A) tail. Nat. Rev. Genet..

[55] Marzluff W.F., Koreski K.P. (2017). Birth and death of histone mRNAs. Trends Genet..

[56] Loll-Krippleber R., Brown G.W. (2017). P-body proteins regulate transcriptional rewiring to promote DNA replication stress resistance. Nat. Commun..

[57] Zhou C., Elia A.E., Naylor M.L., Dephoure N., Ballif B.A., Goel G., Xu Q., Ng A., Chou D.M., Xavier R.J., Gygi S.P., Elledge S.J. (2016). Profiling DNA damage-induced phosphorylation in budding yeast reveals diverse signaling networks. Proc. Natl. Acad. Sci. U. S. A..

[58] Tkach J.M., Yimit A., Lee A.Y., Riffle M., Costanzo M., Jaschob D., Hendry J.A., Ou J., Moffat J., Boone C., Davis T.N., Nislow C., Brown G.W. (2012). Dissecting DNA damage response pathways by analysing protein localization and abundance changes during DNA replication stress. Nat. Cell Biol..

[59] Jamai A., Puglisi A., Strubin M. (2009). Histone chaperone spt16 promotes redeposition of the original h3-h4 histones evicted by elongating RNA polymerase. Mol. Cell..

[60] Morillo-Huesca M., Maya D., Muñoz-Centeno M.C., Singh R.K., Oreal V., Reddy G.U., Liang D., Géli V., Gunjan A., Chávez S. (2010). FACT prevents the accumulation of free histones evicted from transcribed chromatin and a subsequent cell cycle delay in G1. PLoS Genet..

[61] Jeronimo C., Poitras C., Robert F. (2019). Histone Recycling by FACT and Spt6 during transcription prevents the scrambling of histone modifications. Cell Rep..

[62] Wilson M.D., Harreman M., Svejstrup J.Q. (2013). Ubiquitylation and degradation of elongating RNA polymerase II: The last resort. Biochim. Biophys. Acta.

[63] Hobson D.J., Wei W., Steinmetz L.M., Svejstrup J.Q. (2012). RNA polymerase II collision interrupts convergent transcription. Mol. Cell.

[64] Somesh B.P., Reid J., Liu W.F., Søgaard T.M., Erdjument-Bromage H., Tempst P., Svejstrup J.Q. (2005). Multiple mechanisms confining RNA polymerase II ubiquitylation to polymerases undergoing transcriptional arrest. Cell.

[65] Tufegdžić Vidaković A., Mitter R., Kelly G.P., Neumann M., Harreman M., Rodríguez-Martínez M., Herlihy A., Weems J.C., Boeing S., Encheva V., Gaul L., Milligan L., Tollervey D., Conaway R.C., Conaway J.W. (2020). Regulation of the RNAPII pool is integral to the DNA damage response. Cell.

[66] Poli J., Gerhold C.B., Tosi A., Hustedt N., Seeber A., Sack R., Herzog F., Pasero P., Shimada K., Hopfner K.P., Gasser S.M. (2016). Mec1, INO80, and the PAF1 complex cooperate to limit transcription replication conflicts through RNAPII removal during replication stress. Genes Dev..

[67] Hu F., Alcasabas A.A., Elledge S.J. (2001). Asf1 links Rad53 to control of chromatin assembly. Genes Dev..

[68] Emili A., Schieltz D.M., Yates J.R., Hartwell L.H. (2001). Dynamic interaction of DNA damage checkpoint protein Rad53 with chromatin assembly factor Asf1. Mol. Cell.

[69] Tsabar M., Waterman D.P., Aguilar F., Katsnelson L., Eapen V.V., Memisoglu G., Haber J.E. (2016). Asf1 facilitates dephosphorylation of Rad53 after DNA double-strand break repair. Genes Dev..

[70] Yu Y., Eriksson P., Stillman D.J. (2000). Architectural transcription factors and the SAGA complex function in parallel pathways to activate transcription. Mol. Cell. Biol..

[71] Demczuk A., Guha N., Nguyen P.H., Desai P., Chang J., Guzinska K., Rollins J., Ghosh C.C., Goodwin L., Vancura A. (2008). Saccharomyces cerevisiae phospholipase C regulates transcription of Msn2p-dependent stress-responsive genes. Eukaryot. Cell..

[72] Tucker M., Staples R.R., Valencia-Sanchez M.A., Muhlrad D., Parker R. (2002). Ccr4p is the catalytic subunit of a Ccr4p/Pop2p/Notp mRNA deadenylase complex in Saccharomyces cerevisiae. EMBO J..

[73] Badarinarayana V., Chiang Y.C., Denis C.L. (2000). Functional interaction of CCR4-NOT proteins with TATAA-binding protein (TBP) and its associated factors in yeast. Genetics.

[74] Harigaya Y., Parker R. (2012). Global analysis of mRNA decay intermediates in Saccharomyces cerevisiae. Proc. Natl. Acad. Sci. U. S. A..

[75] Anderson J.S., Parker R. (1998). The 3' to 5' degradation of yeast mRNAs is a general mechanism for mRNA turnover that requires the SKI2 DEVH box protein and 3' to 5' exonucleases of the exosome complex. EMBO J..

[76] van Hoof A., Lennertz P., Parker R. (2000). Three conserved members of the RNase D family have unique and overlapping functions in the processing of 5S, 5.8S, U4, U5, RNase MRP and RNase P RNAs in yeast. EMBO J..

[77] Kaluarachchi Duffy S., Friesen H., Baryshnikova A., Lambert J.P., Chong Y.T., Figeys D., Andrews B. (2012). Exploring the yeast acetylome using functional genomics. Cell.

[78] Galdieri L., Zhang T., Rogerson D., Vancura A. (2016). Reduced histone expression or a defect in chromatin assembly induces respiration. Mol. Cell. Biol..

[79] Sherman F. (1991). Getting started with yeast. Methods Enzymol..

[80] Galdieri L., Vancura A. (2012). Acetyl-CoA carboxylase regulates global histone acetylation. J. Biol. Chem..

[81] Coller J. (2008). Methods to determine mRNA half-life in Saccharomyces cerevisiae. Methods Enzymol..

[82] Passos D.O., Parker R. (2008). Analysis of cytoplasmic mRNA decay in Saccharomyces cerevisiae. Methods Enzymol..

[83] Rosebrock A.P. (2017). Analysis of the budding yeast cell cycle by flow cytometry. Cold Spring Harb. Protoc..

